# The Second Annual General Meeting

**Published:** 1917-07-21

**Authors:** 


### THE COLLEGE OF NURSING, Ltd.—(*continued*).

The Poor-Law Nurse.

### THE SECOND ANNUAL GENERAL MEETING.

The second annual general meeting of the College of Nursing, Ltd., was held at the rooms of the Royal Society of Medicine, 1 Wimpole Street, London, W., on Thursday, July 12. The Hon. Arthur Stanley was in the chair, and amongst those present were:—Miss Lloyd Still, R.R.C. (St. Thomas's Hospital), Miss Margaret Hogg (Guy's Hospital), Miss M. Earl (Ancoats Hospital, Manchester), Miss Montgomery (Middlesex Hospital), Miss Pote Hunt (Matron, St. Bartholomew's Hospital, Rochester), Miss E. M. J. Smith, A.R.R.C. (West Didsbury Infirmary, Manchester), Miss Alsop (Kensington Infirmary), Miss Inglis (Shoreditch Infirmary), Miss Girdlestone (late Matron of Crumpsall Infirmary), Miss Sheldon, Lady Superintendent of Guy's Trained Nurses' Institute, Miss Baillie, R.R.C. (Bristol Royal Infirmary), Miss E. Barton, R.R.C. (Matron of Chelsea Infirmary), Miss Graham (Scottish Association of Trained Nurses, Edinburgh), Miss Amy Hughes (late General Superintendent of Queen Victoria's Jubilee Institute for Nurses), Miss E. W. Mowat (Matron, Whitechapel Infirmary), Miss E. M. Musson, R.R.C. (Matron, Birmingham General Hospital), Miss M. E. Sparshott, R.R.C. (Lady Superintendent, Manchester Royal Infirmary), Miss C. E. Vincent, R.R.C. (Lady Superintendent, Leicester Royal Infirmary), Miss Gibson, Colonel D. J. Mackintosh, C.B., M.B. (Superintendent, Western Infirmary, Glasgow), Mr. Comyns Berkeley, M.C., M.D., F.R.C.P. (Honorary Treasurer), and Miss Rundle, R.R.C. (Secretary).

After the notice convening the meeting and the auditors' report had been read by the Secretary, the Chairman, amidst applause, rose to move the adoption of the annual report and accounts.

**The Hon. Arthur Stanley, C.B., M.P.,**

said the members would see from the report that the College had made what might be considered a good start. They were really only quite at the beginning of their work. Different Sub-Committees had been formed, but those Sub-Committees had not yet been able to get at any very serious work, except the Registration Committee, for two reasons. First of all, nurses, who formed the major part of those Sub-Committees, which had got to settle the curriculum and other important matters relating to the nursing profession, were, of course, very busy at present; and, secondly, the constitution of the College had to be definitely settled and all the necessary machinery, such as offices, staff, etc., set up. But having had a good deal to do at different times with the inception of business propositions, he could assure them that the progress made along the dull lines of formation and of offices and all such necessary but tedious work, had got the College very forward during the past year.

The Negotiations with the R.B.N.A.

Mr. Stanley proceeded: We have made no very great effort to enrol nurses, for this reason—that while we are anxious, of course, for them to join the College at the present stage, and those who join it now give us infinitely greater help than if they joined it at a later stage, we have had negotiations, lasting during most of the year, with the Royal British Nurses' Association for the amalgamation of these two bodies. I am glad to say that that amalgamation is in a very fair way of becoming complete, and when it is complete we shall become the Royal British College of Nursing; and we felt that any big effort that we might make had better be deferred until that time, and that to start off and grant all the certificates in the name of the College of Nursing, Ltd., when it is probable that within a few weeks' time we may have to replace those certificates with others of the Royal British College of Nursing, seemed a mere waste of time.

A Society to Speak for Nurses.

But I should like to make it clear that our strength depends, and what we can do in the future will depend, upon the number of our membership. If this College can do what I hope it will do, and what I firmly believe it will do—that is to say, unite in its ranks all those, or practically all those, who have the right to call themselves trained nurses—then we shall be able to speak with a force and with a power that will enable us to get State Registration or anything else in reason that we want. My great difficulty when I first undertook to work in connection with this business was to find any Society that could honestly claim to represent the nurses themselves. There was no such Society, and therefore it became necessary to create one; and that is the reason why we formed the College of Nursing. Our aim and object—the aim and object of every single one of those who have helped me in this work; we have no axe to grind—our one aim and object throughout has been to create a Society which could speak for nurses in the same way as every other profession has got a Society that can speak in its own name. (Hear, hear.)

Over 6,000 Members Already.

Now, of course, the accounts and the report of the College of Nursing only take us up to the end of March. That will account for the fact that the membership roll is smaller than the figures which I have given lately; but the correct figures as now given in the annual report of the Council—that is to say, the figures brought up to date—show that we have received over 7,000 applications for membership. (Applause.) Some of those, of course, were not eligible, but by far the greater part were, and I am

### THE COLLEGE OF NURSING, Ltd.— (*continued*).

very heartily proud to think that our membership roll to-day, when we have only been in existence a little more than a year, already numbers well over 6,000. (Renewed applause.)

### SCOTLAND FORGING AHEAD.

In paragraph 3 of the report we state that "the disadvantages of excessive centralisation have been present to the minds of the Council from the first." Well, if the disadvantages themselves had not been present you could rely upon Scotland and Ireland to make it clear that there were disadvantages. (Laughter.) But from the beginning we have always intended that as far as possible local branches should be set up, working in connection and in close association with the central headquarters here. We have realised, for instance, that it was absurd to think that we here in London could effectively control the nurses in Scotland or Ireland. Well, I need not tell you that they take the same view, and that Scotland and Ireland have both of them formed most satisfactory branches. Scotland, I think, in many ways, has already gone ahead of us here, but at all events it is encouraging to know that those branches are receiving very general support in the two countries to which they belong, and that they are already, though we are not a very old institution ourselves, healthy and promising children. (Applause.)

### THE SUPPLY OF NURSES COMMITTEE.

Mr. Stanley then referred to the not unimportant part played by the College in the constitution in its final form of the Committee which was set up by the War Office to inquire into the supply of nurses, which, as originally constituted, had no nurse member whatever on it, and only one lady, she being the Commandant-in-Chief of the V.A.D.s. As the net result of their joint efforts no less than six representatives from the College Council were appointed on to that Committee.

### STATE REGISTRATION.

In dealing with the position in this regard, Mr. Stanley said: "My experience during the past eighteen months in which I have had to deal with this work is that it is very difficult to make any remarks on this subject, either in public or in private, that cannot be misrepresented—(laughter)—but I will endeavour to make our position as clear as possible. From the very beginning—I say from the very beginning, but I think my first letter that was published said that I had some doubt as to whether there was a universal demand for State Registration, and, of course, I very soon had to realise that there was a universal demand—we have always held, and always worked on the understanding, that one of the first objects which the College set out to attain was the State Registration of nurses. (Hear, hear.) Now, in order that there may be no doubt about this, let me state once more, if there is anybody who doubts or who really pretends to doubt—let me state once more that the College of Nursing and the Council of the College of Nursing are absolutely wholehearted in their intention to obtain State Registration, and are perfectly confident that they will get it. (Applause.) Well, when we had been at work for some little time we thought it might be advisable to see whether those who were also working for State Registration could not be brought together, so that we could promote an agreed Bill. We had several conferences with representatives of the Central Committee for State Registration of Nurses. We got on very well—so far, indeed, that at the last conference which we had—which was some time, I think, in July last year—I thought, and I believe that practically everybody else present there thought, that practically all the questions had been settled, and that we had got an agreed Bill.

### HOW THE NEGOTIATIONS WERE BROKEN OFF.

However, in September, over a point of no great importance, the Central Committee for State Registration thought wise to break off negotiations altogether, and to say that they would have nothing to do with the Bill which we had practically agreed, but would go ahead and promote their own Bill. The point which they made their reason for separating themselves from us was a very simple one. We had agreed that the Central Committee for State Registration should be empowered to appoint a certain number of members to the first General Nursing Council under the Bill which we were going to promote. The College of Nursing reserved to itself the right of appointing the same number of representatives. But we held, and I still hold, that it was very advisable that in the Bill itself the names of those representatives should be given, and we hold that opinion for two reasons. The first is that, under the Bill, that Provisional Council is to hold office probably for two years, which, of course, will be a very important time, as during that period so many of the rules and regulations will be made, although, of course, those rules and regulations could be altered afterwards. We held, therefore, that it was very important that the nurses should know who were going to govern them for the first two years of State Registration. I still think that that is a very important point.

### INDIVIDUALS OR SOCIETIES?

But there was a second reason why we wished for the names. If you do not put in the names, you have to put in that certain bodies, such as the Central Committee for State Registration, and such as the College of Nursing, are appointing so many representatives. The minute you put that in you are perfectly certain to get all kinds of small societies from all over the place springing up and trying to get Parliament to give them similar rights of representation. Well, it would be quite impossible to have, it might be, twenty or thirty societies all nominating representatives. We thought, therefore, that infinitely the simplest thing was to put straightforwardly in the Bill exactly those representatives who were going to govern the nursing profession during the first two years under State Registration. Now, as I say, it is not a point that there was any necessity to split over. Those of us who really wish to have State Registration, those of us who really wish to obtain State recognition for the great nursing profession, are not going to be led aside or hindered by any such points as this. (Applause.) The whole thing was a question of expediency, and nothing else.

### THE PRESENT POSITION.

The position at this moment is this: I happened by chance to see Major Chapple—who, as you know, was the introducer of the Bill brought forward for some years past by the Central Committee for State Registration—I happened to see him a few days ago, and I told him that the position of the College of Nursing is now what it always has been—we are always willing to enter into conferences or to discuss with any society that can properly claim in any way to represent nurses; we are always ready and willing to discuss with them the terms of any Bill that we may wish to bring in. All that we have in our minds, all that we want to do in this matter, is to get a State Registration Bill, and to get it in the terms that will be best for the nurses themselves. (Applause.)

After reviewing the events which led the members of the Royal British Nurses' Association to approve the resolution to amalgamate with the College of Nursing, Mr. Stanley dealt with the question of Poor-Law nurse-training schools.

### THE POOR-LAW ASSOCIATIONS.

You will also, I think, have seen—those of you who have followed the fortunes of the College of Nursing during the past year—that we have had meetings with representatives of the two Poor-Law Associations. You also may have gathered from some of the reports in the Poor-Law papers that those Associations are not entirely pleased with us. Our position there, again, is perfectly simple. We recognise that there are a large number of Poor-Law training-schools that come under the definition of schools recognised by the College of Nursing. We realise that there are a

### THE COLLEGE OF NURSING, Ltd.—(*concluded*).

large number of nurses trained in those schools, and that those nurses are obviously entitled to representation on the Council of the College of Nursing. Of course, in two years' time, when the Council of the College of Nursing, instead of being nominated, becomes entirely elective—because in two years' time we all go out of office, and you have it in your power to keep us out of office and to put in anybody else you like—we all recognise that it is right that the Poor-Law nurses should have their fair share of representation on the Council. But what we have also got to bear in mind is this: that it is those nurses who help us now, those nurses whose names appear on the Register now, who are really doing the pioneer work, and it is only right that the representation of those nurses on the Council should be in proportion to the numbers of the nurses on the Register of the College.

### Representation on the Council.

What I mean is this—that if the Poor-Law nurses on the existing Register of the College numbered, let us say, half the total number of nurses on the Register, it would be right that they should have something like half the representation on the Council. But they do not number that, and we must look to the actual people who are on our Register. I think I am right in stating that according to those numbers the proper representation of the Poor-Law nurses on the Council of the College of Nursing would be four, instead of three as it is at present. Well, we shall take the earliest possible opportunity—I know I can speak for my colleagues as well as myself—of seeing that the Poor-Law nurses whose names are on our Register receive their proper representation on the Council. (Applause.)

### The College Council.

Well, ladies and gentlemen, I must not detain you too long. I have got a few questions here, and I shall give those of you who want to ask further questions an opportunity of doing so. I want just to repeat what I said at the beginning. I have now a fairly intimate acquaintance with those with whom I have been working for the last eighteen months, and with those who form the Council of the College of Nursing. I see it stated by some of those who are not friendly to the movement that these people have various axes to grind—that they are doing things for their own ends—that they are not thinking only of the nurses. Well, I say from my own intimate knowledge that that is absolutely untrue. (Applause.) I have never worked with a Council who were more united, or who were more wholehearted than they are in the task to which they have set their hands. Occasionally we have been accused, I think, of changing our opinions. That, in the formation of a great College such as this, is absolutely necessary. No human being could have sat down two years ago and put down on paper, in black and white, exactly what the constitution of a great body such as this should be. What we have done, I think, has been to take advantage of—we certainly have listened to—every suggestion—every reasonable suggestion that was brought to our notice. I think that sometimes even the nurses themselves do not quite realise the magnitude of the task. Here was a great profession, practically without any organisation. Now it has very seldom happened that anybody has had to begin to organise a profession that certainly counted already 30,000 or 40,000 members. All the great Colleges of the world, all the great institutions, have sprung from very small beginnings. But we began ours rather in the same way in which this country began in the great war. We began faced with the greatest possible questions, with questions of intense importance to a large number of nurses. We had to begin from the very beginning; we had to build up the whole of our organisation; we had to deal with problems which as a rule do not come before a College of this kind until many years after the beginning of its existence. (Hear, hear.) Well, ladies and gentlemen, if you think that we have made a good beginning, if you think that we have shown ourselves wholehearted in our endeavour to do what is best for the nursing profession, then I ask you to continue to give us the support which you have been kind enough to give us up till now, and to do your best to spread among your colleagues and friends the knowledge of the real aims and objects of the College of Nursing, Limited. (Applause.)

I beg to move that the annual report and accounts be adopted. I will ask Miss Musson to second that, and then I shall be glad to answer any questions.

### Miss Musson, R.R.C.

It gives me very much pleasure to second the adoption of the report. I have not come here prepared to make a speech, but I should like to say that whenever I have spoken in this room it has always been on the subject of State Registration. I still hold that State Registration is the most important matter before us, and you will see from the report that it is considered the most important piece of work yet before the College. There is another piece of work which also lies, in the future, I hope, in the hands of the College, and that is the building up of nursing education, which is, as we all know, very, very small now. Mr. Stanley used the phrase just now, "Even the nurses hardly understand." I would like to say that it is very important that nurses should begin to think a little bit more for themselves. We are sometimes accused of acting for our nurses. I find that my great difficulty is to make my nurses do anything but thrust me forward to speak for them. (Laughter.) I do hope that the nurses will take an interest in this College for themselves, and not confine themselves merely to thrusting forward someone else to speak for them.

Mr. Stanley then put to the meeting the resolution adopting the report and accounts for the year 1916, which was carried unanimously. Miss A. Baillie proposed the re-election of Messrs. Barton, Mayhew and Co. as auditors for the year 1918, their remuneration to be fixed by the council, which was carried.

### Miss Amy Hughes.

Before the meeting breaks up I venture to propose a hearty vote of thanks to Mr. Stanley for being our chairman, and for the most interesting and helpful address which he has given to us this afternoon. I am sure that those nurses who wished to know what was going on in regard to the College of Nursing will feel that they can go away and spread the information they have acquired amongst their friends and colleagues with every confidence of good results accruing.

This resolution was carried amidst loud applause, and The Chairman then said: Thank you very much, ladies and gentlemen, for the way in which you have passed this vote of thanks, and you, ladies, for the way in which it has been proposed and seconded. It is a very great pleasure to have had this meeting to-day, and to get the encouragement which you have given us. I feel absolutely certain that we are going to get what we want, but still it is always very pleasant to be encouraged on the way. (Applause.)

